# The Virulence Factor LLO of *Listeria monocytogenes* Can Hamper Biofilm Formation and Indirectly Suppress Phage-Lytic Effect

**DOI:** 10.3390/foods14152554

**Published:** 2025-07-22

**Authors:** Banhong Liu, Mei Bai, Wuxiang Tu, Yanbin Shen, Jingxin Liu, Zhenquan Yang, Hongduo Bao, Qingli Dong, Yangtai Liu, Ran Wang, Hui Zhang, Liangbing Hu

**Affiliations:** 1 Jiangsu Key Laboratory for Food Quality and Safety-State Key Laboratory Cultivation Base of MOST, Jiangsu Academy of Agricultural Sciences, Nanjing 210014, China; 2 School of Food and Biological Engineering, Shaanxi University of Science and Technology, Xi’an 710021, China; 3 School of Food and Biological Engineering, Jiangsu University, Zhenjiang 212013, China; 4 School of Food Science and Engineering, Yangzhou University, Yangzhou 225001, China; 5 School of Health Science and Engineering, University of Shanghai for Science and Technology, Shanghai 200093, China; qdong@usst.edu.cn (Q.D.);

**Keywords:** *Listeria hemolysin*, biofilm, phage susceptibility, transcriptomics

## Abstract

*Listeria monocytogenes* is a life-threatening bacterial foodborne pathogen that can persist in food-processing facilities for years. Although phages can control *L. monocytogenes* during food production, phage-resistant bacterial subpopulations can regrow in phage-treated environments. In this study, an *L. monocytogenes hly* defective strain, NJ05-Δ*hly*, was produced, which considerably regulated the interactions between *L. monocytogenes* and phages. Specifically, we observed a 76.92-fold decrease in the efficiency of plating of the defective strain following infection with the *Listeria* phage vB-LmoM-NJ05. The lytic effect was notably diminished at multiplicities of infection of 1 and 10. Furthermore, the inactivation of LLO impaired biofilm formation, which was completely suppressed and eliminated following treatment with 10^8^ PFU/mL of phage. Additionally, phages protected cells from mitochondrial membrane damage and the accumulation of mitochondrial reactive oxygen species induced by *L. monocytogenes* invasion. Transcriptomic analysis confirmed these findings, revealing the significant downregulation of genes associated with phage sensitivity, pathogenicity, biofilm formation, and motility in *L. monocytogenes*. These results underscore the vital role of LLO in regulating the pathogenicity, phage susceptibility, and biofilm formation of *L. monocytogenes*. These observations highlight the important role of virulence factors in phage applications and provide insights into the potential use of phages for developing biosanitizers.

## 1. Introduction

*Listeria monocytogenes* is a major intracellular foodborne bacterial pathogen that can cause invasive listeriosis, a human systemic infection, particularly in vulnerable groups such as pregnant women, fetuses, newborns, seniors, and immunocompromised individuals. This pathogen can survive at lowtemperatures and contaminate refrigerated food products [1,2]. Invasive listeriosis is associated with a high mortality rate (20–30%), Consequently, it ranks the third leading cause of death from foodborne infections in the US [3,4]. The presence of this pathogen in food production environments has caused multiple listeriosis outbreaks in different food products [5].

Although *L. monocytogenes* can be inactivated by thermal treatments applied to processed foods, cross-contamination may arise from equipment and environmental sources owing to biofilm formation, which threatens public health and challenges control measures [6,7]. *L. monocytogenes* strains in biofilms are less susceptible than planktonic cells to the industrially used quaternary ammonium compound benzalkonium chloride. The minimum inhibitory concentration is 64 times higher for sessile cells than that for planktonic cells [8]. Furthermore, tetracycline resistance (since 1992) and fluoroquinolone resistance (since 2014) have emerged in food-source *L. monocytogenes* [9,10]. Foodborne isolates of *L. monocytogenes* that are highly resistant to ceftazidime, ciprofloxacin, and lincomycin have also been documented [11].

The extensive use of antibiotics has led to the emergence of antimicrobial-resistant bacteria. Compared to conventional antimicrobial agents and methods, *L. monocytogenes* phages are regarded as a promising antimicrobial strategy in the food industry, as they can eliminate pathogenic bacteria [12]. A previous study demonstrated that the *Listeria* phage SH3-3, which exhibits an effective minimum inhibitory concentration, can inhibit the formation of dense, net-like structures characteristic of *L. monocytogenes* biofilms [13]. Moreover, they do not alter the organoleptic or nutritional characteristics of food [14]. Phages bind to bacterial receptors via receptor recognition mechanisms. The role of wall teichoic acid-associated N-acetylglucosamine, rhamnose, and *glcV* coding products (glycosyl transferases) as phage receptors in *L. monocytogenes* [15,16,17] has been previously established. However, no reports are available on the interactions between virulence factors and phages.

One of the key virulence factors of *L. monocytogenes* is Listeriolysin O (LLO), which is encoded by *hly* located on the first pathogenicity island of *L. monocytogenes* (LIPI-1) [18]. The LLO function was initially characterized in *Listeria* escaping from the phagosome under acidic conditions [19]. However, several studies have now observed that this crucial virulence factor also displays activity at neutral pH and acts on cells before bacterial entry [20,21]. Inactivation of LLO leads to a loss of hemolytic activity and considerably reduces virulence in murine models [22,23]. However, its regulatory role in self-initiated biofilm formation and phage susceptibility has rarely been investigated. This study used food-derived *L. monocytogenes* as the model organism. We constructed an LLO-deletion mutant using homologous recombination and characterized its biological properties, including growth kinetics, motility, cell adhesion, and invasion capacity. These investigations have elucidated the role of the virulence factor LLO in *L. monocytogenes* growth, virulence expression, and phage susceptibility. These findings provide new targets for the prevention and control of *L. monocytogenes.*

## 2. Materials and Methods

### 2.1. Bacteria, Phage, and Growth Conditions

*L. monocytogenes* strain NJ05 (1/2a) was isolated from pig hind-leg meat in a slaughterhouse. Briefly, 25 g of slaughterhouse samples underwent sequential secondary enrichment in Half-Fraser broth (Merck, Hesse, Germany), followed by Full-Fraser broth (Merck, Hesse, Germany). Post-enrichment cultures were screened using the dual-plate method on Oxford and Palcam agars (Merck, Hesse, Germany). After purification on Tryptone Soy agar supplemented with 0.6% yeast extract (Merck, Hesse, Germany), *Listeria* spp. identification was confirmed through Gram staining, catalase testing, motility assessment, and API *Listeria*^®^ (BioMérieux, Marcy-l’Étoile, France) analysis. Subsequently, *L. monocytogenes* isolates were serotyped using O and H antigen agglutination assays [11]. The shuttle vector pKSV7 was kindly provided by Dr. Guoqiang Zhu (Yangzhou University, Yangzhou, China). The plasmid pIMK2, used to complement the defective strain, was supplied by Dr. Yuelan Yin (Yangzhou University, Yangzhou, China). *Listeria* phage vB-LmoM-NJ05, with wide lytic activity to *Listeria* spp., including *L. monocytogenes*, *L. innocua*, and *L. welshimeri* was previously isolated from sewage at a pig farm in Jiangsu, China [24]. Briefly, slaughterhouse sewage was centrifuged and filtered at 4 °C, and the supernatant was incubated overnight with *L. monocytogenes* strain NJ05 at 30 °C. The filtered supernatants were subjected to spot testing and double-layer plaque assays. Phage-containing plaques were excised, suspended in phosphate-buffered saline (PBS, pH 7.4), centrifuged, filtered, and stored as “spot filtrate” at 4 °C. Phage suspensions were re-incubated with NJ05 (30 °C, 18 h) for purification. *L. monocytogenes* strains were streaked on Brain Heart Infusion (BHI) agar or in tryptic soy agar (TSB, Qingdao Hope Biol-Technology Co., Ltd., Qingdao, China), and incubated for 20 h at 30 °C. All strain stocks were stored at −80 °C in BHI broth, supplemented with 20% glycerol. The phages proliferate by infecting the host strain NJ05 under incubation at 30 °C for approximately 20 h [13].

### 2.2. Generation of L. Monocytogenes Mutant Strain and Complementation Studies

PCR primers were designed based on the nucleotide sequence of *hly* (NC_003210.1) [2]. All primers used in this study were synthesized by Sangon Biotech Co., Ltd. (Shanghai, China) Fragments (372 bp) comprising the upstream and downstream regions of *hly* were generated using gene splicing by overlap extension (SOE) PCR. The *hly deletion* sequence, designated Δ*hly*, was cloned into a pUC57 vector and subsequently sequenced by Sangon Biotech (Shanghai) Co., Ltd. The Δ*hly* fragment was purified and digested with restriction enzymes (Thermo Fisher Scientific Inc. Shanghai) *Bam*HI and *Kpn*I. This linear fragment was then cloned into the corresponding sites of temperature-sensitive pKSV7 shuttle vector. Electro-competent *L. monocytogenes* strain NJ05 was transformed with the construct and subjected to temperature-dependent allelic exchange. The in-frame deletion of *hly* was confirmed by PCR using the primer pair *hly*-F3 and *hly*-R3.

To complement the Δ*hly* mutant with *hly*, oligonucleotide primers *hly*-F1 and *hly*-R1 (Table 1) were used to amplify the complete *hly* open reading frame from the genomic DNA of *L. monocytogenes* NJ05. The amplified products were gel-purified, sliced with *Xho*I and *Bam*HI, and cloned into pUC57 cut with *Xho*I and *Bam*HI, followed by sub-cloning into the pIMK2 vector cut with *Xho*I and *Bam*HI [25]. The complemented strain was verified using RT-PCR and hemolytic assays and designated NJ05-Δ*hly*::*hly.*

### 2.3. Hemolytic, Growth, and Swimming Characteristics of the Strains

To determine the function of *hly* in wild-type *L. monocytogenes*, the biological characteristics of the parental strain, an *hly deletion* strain, and a complemented strain were examined. *L. monocytogenes* strains NJ05, NJ05-Δ*hly*, and NJ05-Δ*hly*::*hly* were inoculated into BHI liquid medium at 37 °C and 180 rpm until they reached the logarithmic phase (OD_600nm_ 0.6). Subsequently, they were inoculated at 1% concentration into fresh BHI liquid medium and incubated at 37 °C. The optical density (OD_600nm_) was measured using a microplate reader every 30 min. Moreover, the logarithmic strains were plated onto 5% sheep blood agar and incubated at 37 °C for 24 h to assess the extent of hemolysis [26].

The abovementioned strains were incubated overnight at 30 °C with shaking at 180 rpm. The cultures were adjusted to an OD_600_ of approximately 1.0, and each culture (5 μL) was stab-inoculated into semisolid TSA plates (0.3% agar). The plates were then incubated at 30 °C for 48 h, after which the motility diameters were observed and measured using a metric ruler [27].

### 2.4. Transcriptome Analysis

To comprehensively understand the metabolism associated with *hly* in *L. monocytogenes*, a comparative transcriptomic analysis was performed between the wild-type strain NJ05, the mutant NJ05-Δ*hly,* and the complemented strain NJ05-Δ*hly*::*hly*. Logarithmic phase cultures of *L. monocytogenes* NJ05, NJ05-Δ*hly*, and NJ05-Δ*hly*::*hly* were incubated overnight at 30 °C with shaking at 180 rpm, collected, and rapidly frozen in liquid nitrogen. Total RNA was extracted using the TRIzol Total RNA Extractor kit (Invitrogen, Carlsbad, CA, USA). The integrity of RNA was assessed using an Agilent 2100 Bioanalyzer (Agilent Technologies, Munich, Germany). A cDNA library was constructed from high-quality RNA samples, which were also evaluated using the Agilent 2100 Bioanalyzer, and the library concentration was determined using RT-PCR. Data quality control was performed using the Fast-QC software(v0.11.9). Clean reads were obtained by filtering out low-quality reads and those containing adapter contamination, which were subsequently mapped to the predicted transcripts of *L. monocytogenes* EDG-e. Differentially expressed genes (DEGs) were employed to analyze the differences in gene expression, selecting DEGs with |log2FoldChange| > 1 and *p*-value < 0.05. DEGs were visualized using a volcano plot and subjected to Gene Ontology (GO) and Kyoto Encyclopedia of Genes and Genomes (KEGG) functional enrichment analyses to identify significant enrichments in GO terms or metabolic pathways. All data reported are averages from three independent replicates [28].

### 2.5. Influence of Environmental Factors on Biofilm Formation

Biofilm assays were performed on polystyrene microplates, following the procedures outlined by Zhou et al. [11], with some modifications. Briefly, *L. monocytogenes* was cultured for 24 h and diluted to a concentration of 10^8^ CFU/mL. For biofilm formation, 0.1 mL of the diluted suspension (10^7^ CFU/mL) was inoculated into each well of 96-well polystyrene microplates (Corning Incorporated, Corning, New York, NY, USA) and incubated statically at 37 ± 0.5 °C for 48 h. After incubation, the microplates were washed with sterile phosphate-buffered saline (PBS; pH 7.2), fixed with methanol, and stained with 0.1% crystal violet for 15 min. The plates were then dried and resolubilized with 33% (*v*/*v*) glacial acetic acid solution. The optical density of each well was measured at 590 nm using a microtiter plate reader (Tencan Austria GmbH, Infinite 200 Pro, Groedig, Austria). The number of *L. monocytogenes* strains was enumerated using the plate-counting method to assess the different capabilities of biofilm formation.

To examine the effects of various environmental conditions on biofilm formation, the microplates were incubated at different temperatures (25 °C, 30 °C, and 37 °C), with varying concentrations of BHI broth (25%, 50%, 75%, and 100%), sodium chloride (0%, 0.25%, 0.5%, 1%, 2%, 4%, and 8%), and glucose (0%, 0.25%, 0.5%, 1%, 2%, 4%, and 8%) for 48 h. The ability of biofilm formation was determined using the crystal violet staining method [11].

### 2.6. Adhesion and Invasion

The adhesion and invasion capabilities of the *L. monocytogenes* strains were evaluated using the RAW264.7 mouse macrophage cell line at a multiplicity of infection (MOI) of 100. For the adhesion assay, the experimental cells were infected for 1 h, washed, and lysed with 0.1% Triton X-100. Adherent bacteria were quantified by plating on BHI agar. For the invasion assay, *L. monocytogenes* strains were prepared and inoculated similarly. An hour post-infection, the cells were washed, treated with 100 μg/mL gentamicin, and incubated at 37 °C for 1 h and 30 min, followed by washing again and lysis with 0.1% Triton X-100 to enumerate viable bacteria [27].

To assess the effect of the phage on cell adhesion and invasion, the cells were incubated with 0.1 mL of phage vB-LmoM-NJ05 (10^8^ plaque-forming units (PFU)) for 24 h at 37 °C. Following incubation, the cells were washed thrice with PBS and infected with *L. monocytogenes* at an MOI of 100 for approximately 1.5 h. Viable bacteria were quantified as described previously [27].

### 2.7. Phage Susceptibility and Efficiency of Plating (EOP)

Phage propagation was conducted as previously described by Zhang et al. [29]. Briefly, the phage lysate was filtered through a 0.22-μm membrane and stored at 4 °C. The phage titer was subsequently determined, and an EOP assay was performed according to the methodology outlined by Tian et al. [30]. Briefly, phage vB-LmoM-NJ05, serially diluted (1 × 10^9^ PFU/mL), was combined with *L. monocytogenes* strains NJ05, NJ05-Δ*hly*, and NJ05-Δ*hly*::*hly*. The phage titers in each strain were quantified using a double-layer agar assay. EOP was calculated as the ratio of the phage titer in the test strain to that in the reference strain (NJ05), with results averaged from three independent assays.

### 2.8. Adsorption and Growth Curve

The adsorption of phages to their host strains is the initial step in their life cycle and is critical for lytic activity. In this study, the adsorption of phage vB-LmoM-NJ05 to the mutant NJ05-Δ*hly* was determined by assessing the free PFUs in the medium following incubation with *L. monocytogenes* cells [13]. Phage suspension with a concentration of 10^7^ PFU/mL was combined with a fresh culture of *L. monocytogenes* at a concentration of 10^9^ CFU/mL to achieve an MOI of 0.01. The phage-bacterial mixture was incubated at 37 °C for 30 min, and samples were collected at 5-min intervals during incubation. The suspension was then centrifuged at 12,000 rpm for 10 min. The titer of free phages (PFU/mL) in the supernatant was quantified, and the average results from at least three experiments are presented.

Subsequently, fresh *L. monocytogenes* cultures with an optical density (OD_600nm_) of 1 were inoculated with phages to achieve MOIs of 1 and 10. Bacterial growth was monitored hourly by measuring OD_600nm_ after 1 to 9 h of shaking at 37 °C. A control, consisting of bacterial growth without phage addition, was evaluated over the same 9-h period. Each experimental condition was replicated thrice, and the average data, along with standard deviations, were reported [13].

### 2.9. Inhibition and Removal of Biofilms by L. monocytogenes Phage

*L. monocytogenes* strains were inoculated into 96-well polystyrene microplates and cultured for 24 h. Subsequently, 0.1 mL of phage at varying concentrations was added to each well, followed by static incubation at 37 ± 0.5 °C for 12 h. Biofilm formation was assessed in the same way. To evaluate the efficacy of phages in biofilm removal, after 48 h of biofilm formation, 0.1 mL of phage at different concentrations was added to each well, and the plate was incubated statically at 37 ± 0.5 °C for an additional 12 h. Negative controls without phage addition were included for comparison. Crystal violet assay was used to quantify biofilm formation, and the optical density of each well was measured at 590 nm using a microtiter plate reader (Tencan Austria GmbH, Infinite 200 Pro, Groedig, Austria).

### 2.10. Mitochondrial Membrane Potential and Reactive Oxygen Species (ROS) of Mutants NJ05-Δhly

The mitochondrial membrane potential of RAW264.7 cells was assessed after infection with *L. monocytogenes* strains. RAW264.7 cells were seeded into six-well plates and treated with phages or stimulated with different *L. monocytogenes* strains, as specified. The cells (10^6^ cells/well) were incubated for 24 h with 10^8^ PFU/mL phage vB-LmoM-NJ05. Next, the cells were washed thrice with PBS and infected with *L. monocytogenes* at an MOI of 50 for approximately 1.5 h. To evaluate the mitochondrial membrane potential, the mitochondria were stained with Mito-Tracker Red CMXRos (100 nmol/L) and 4′,6-diamidino-2-phenylindole (DAPI, 1 μg/mL), according to the manufacturer’s instructions. After 10 min incubation at 37 °C, the cell supernatant was discarded, and the cells were washed with PBS. The stained cells were subsequently analyzed using a fluorescence microscope (Bio-Rad, Hercules, CA, USA).

ROS play a critical role in the innate immune response against intracellular bacteria. For the measurement of ROS, the culture medium was removed, and the cells were washed with PBS before staining with DAPI (1 μg/mL) and MitoSOX (1 μmol/L). After 10 min of incubation at 37 °C, the cell supernatant was removed, and the cells were washed with PBS. The levels of superoxide as ROS were analyzed using a fluorescence microscope (Bio-Rad, Hercules, CA, USA).

### 2.11. Statistics and Reproducibility

All assays were independently repeated with at least three biological replicates, and the experimental data are presented as the mean ± standard deviation. Student’s *t*-test was employed to analyze the significance of differences between the two groups, while a one-way analysis of variance (one-way ANOVA followed by Waller-Duncan) was performed for an overall comparison among multiple groups (SPSS, version 25.0). *p* < 0.05 was considered to indicate statistical *significance*.

## 3. Results

### 3.1. Construction of the Mutant L. monocytogenes Strain NJ05-Δhly

**Figure 1:**
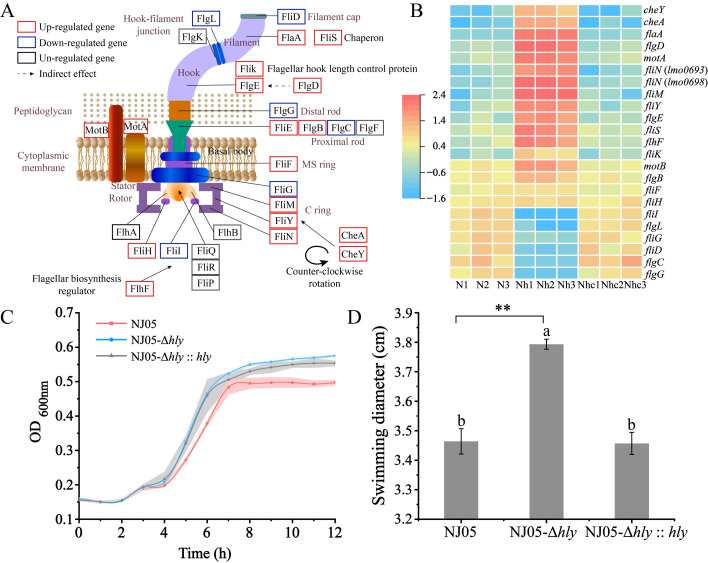
Enhanced swimming capacity of NJ05Δ*hly*. (**A**) Flagellar structure of *L. monocytogenes* and gene products involved in flagellar assembly and regulation based on KEGG mapping (Ko02030 and Ko02040). (**B**) Heatmap illustrating the upregulated and downregulated genes; gene names are indicated. Red and blue shades represent high and low gene abundance, respectively. N1, N2, and N3 represent three repetitions of NJ05; Nh1, Nh2, and Nh3 represent three repetitions of NJ05-Δ*hly*; and Nhc1, Nhc2, and Nhc3 represent three repetitions of NJ05-Δ*hly*::*hly*. (**C**) Growth curves of mutant NJ05-Δ*hly* and wild-type NJ05 were included as a control. (**D**) Mutants and WT were grown on swimming plates (0.3% agar). The swimming ability of NJ05-Δ*hly* was enhanced compared to that of the WT. Different letters in the figure indicate significant differences (*p* < 0.05, one-way ANOVA followed by Waller-Duncan). ** *p* < 0.01 compared with the wild-type NJ05.

A defective mutant strain, NJ05-Δ*hly*, and its complemented strain, NJ05-Δ*hly*::*hly*, were constructed (Supplementary Figure S1). As expected, deletion of LLO in strain NJ05-Δ*hly* considerably reduced its hemolytic activity on blood agar. However, hemolytic activity was restored in the complemented strain NJ05-Δ*hly::hly*. In addition, the growth pattern of the defective mutant NJ05-Δ*hly* was consistent with that of the parental strain (Figure 1C).

### 3.2. Transcriptomic Analysis of Differential Gene Expression

A volcano plot was generated using R, as shown in Supplementary Figure S2. A total of 322 genes were significantly upregulated, and 1896 were significantly downregulated. GO analysis indicated that the DEGs were significantly enriched in DNA repair, cellular response to stress, and protein metabolic processes. The absence of *hly* altered cellular components, primarily affecting DNA repair mechanisms, which in turn influenced cellular proteins and metabolic processes. Furthermore, KEGG analysis revealed metabolic pathway changes resulting from the deletion of *hly*. The top 20 KEGG pathways with the most significant enrichment were selected. Peptidoglycan biosynthesis, purine metabolism, aminoacyl tRNA biosynthesis, cysteine and methionine metabolism, and D-amino acid metabolism were among the metabolic pathways that were significantly enriched.

### 3.3. Upregulation of Genes Encoding Chemotaxis and Flagellar Assembly in the Mutant NJ05-Δhly

The flagellum of *L. monocytogenes* comprises a basal body, hook, and filament. The basal body functions as the flagellar motor, supplying the power needed for rotation, whereas the protein export apparatus secretes more distal components. The hook, which is connected to the basal body, serves as a flexible universal joint of the flagellum. The filament, which is composed of flagellin, acts as a propeller [31,32]. The interaction between the complex proteins (FliG, FliM, FliY, and FliN) constituting the flagellar motor switch and the *cheY* gene changes the flagellar rotation from clockwise to counter-clockwise. This transition induces a shift from tumbling to smooth swimming behavior (Figure 1A) [33]. As illustrated in Figure 1B, the expression of 15 of the 26 genes involved in flagellar assembly was upregulated in the mutant NJ05-Δ*hly*. These genes included flagellar motor switch regulatory genes (*fliM*, *fliY*, and *fliN*) and motility genes (*motA*). Swimming motility experiments were conducted to evaluate the motility of the mutant NJ05-Δ*hly*, which revealed that the diffusion distance was considerably greater than that of the parental strain (Figure 1D). This finding is consistent with the changes observed in the expression of the aforementioned genes.

### 3.4. Reduction of Biofilm Formation in the Mutant NJ05-Δhly

**Figure 2:**
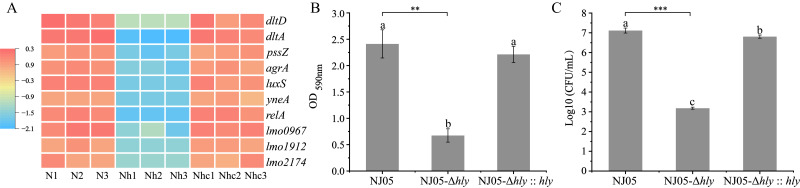
Biofilm formation by mutant NJ05-Δ*hly*. (**A**) Heatmap analysis of biofilm-related gene expression. The legend for the diagram is shown in Figure 1. (**B**) Biofilm assays were conducted in 96-well polystyrene microplates. Serial dilutions were prepared in BHI and inoculated with 0.1 mL of each well. The optical density of each well was measured at 590 nm. (**C**) The number of *L. monocytogenes* strains was counted after biofilm formation. Values corresponding to the numbers indicate the means of three independent experiments. Different letters in the figure indicate significant differences (*p* < 0.05, one-way ANOVA followed by Waller-Duncan). ** *p* < 0.01; *** *p* < 0.001 when compared with the wild-type NJ05.

Genes linked to initial attachment, such as *dltD*, *dltA*, and *rmlA*, and those regulating flagella- and toxin-related gene expression, were downregulated in the mutant strain NJ05-Δ*hly*. Furthermore, genes associated with biofilm formation, such as *pssZ* and *relA*, were downregulated. This included the downregulation of regulatory genes for certain signaling molecules, including *agrA* and *luxS* (Supplementary Figure S3 and Figure 2A).

**Figure 3:**
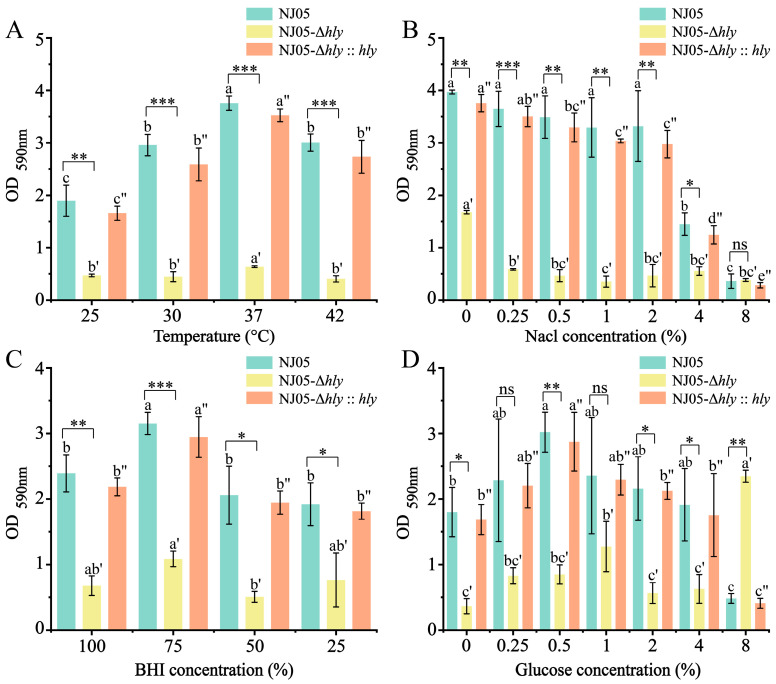
Effect of stress conditions on biofilm formation. The effects of (**A**) different temperatures (25 °C, 30 °C, 37 °C, and 42 °C), (**B**) BHI concentration (25%, 50%, 75%, and 100%), (**C**) NaCl concentration (0, 0.25%, 0.5%, 1%, 2%, 4%, and 8%), and (**D**) glucose concentration (0, 0.25%, 0.5%, 1%, 2%, 4%, and 8%) on biofilm formation. The optical density of each well was measured at 590 nm after 48 h of incubation. Different letters in the figure indicate significant differences and the letters with primes indicate different groups (*p* < 0.05, one-way ANOVA followed by Waller-Duncan). * *p* < 0.05; ** *p* < 0.01; *** *p* < 0.001 when compared with the wild-type NJ05; ns, no significance.

The biofilm-forming ability of the mutant strain NJ05-Δ*hly* was investigated under various environmental conditions. Crystal violet staining indicated that the biofilm-forming capability decreased significantly by 70.83% (*p* < 0.01) after the deletion of *hly* (Figure 2B). After biofilm formation, a 3.93 ± 0.08 log reduction in the bacterial population was observed within the biofilm (Figure 2C). Therefore, *hly* deletion and biofilm formation appear to be strongly correlated. In addition, the biofilm-forming ability of the mutant NJ05-Δ*hly* was examined under different environmental conditions. The results are shown in Figure 3. The wild-type strain NJ05, mutant NJ05-Δ*hly*, and complemented strain NJ05-Δ*hly*::*hly* exhibited a gradual increase in biofilm formation between 25 °C and 37 °C, followed by a decline at 42 °C (Figure 3A). The biofilm-forming ability of *L. monocytogenes* NJ05-Δ*hly* was significantly lower than that of the wild-type strain, NJ05, and the complemented strain. Moreover, the decreased ability of *L. monocytogenes* NJ05-Δ*hly* to form biofilms was independent of temperature, BHI medium, and NaCl concentration (Figure 3A–C), indicating that these factors do not directly influence the process. The addition of glucose exerted a pronounced effect on the biofilm-forming ability of NJ05-Δ*hly*; at an 8% glucose concentration, the OD_590nm_ was 2.32 ± 0.09. This finding implies a potential metabolic interaction wherein glucose enhances biofilm formation in the absence of *hly*. This raises intriguing questions about how carbohydrate availability might influence pathological traits, potentially aiding in the development of measures to control biofilm formation in industrial and healthcare settings. This compensatory mechanism warrants further investigation.

### 3.5. Reduction of Adhesion and Invasion Rates in the Mutant NJ05-Δhly

**Figure 4:**
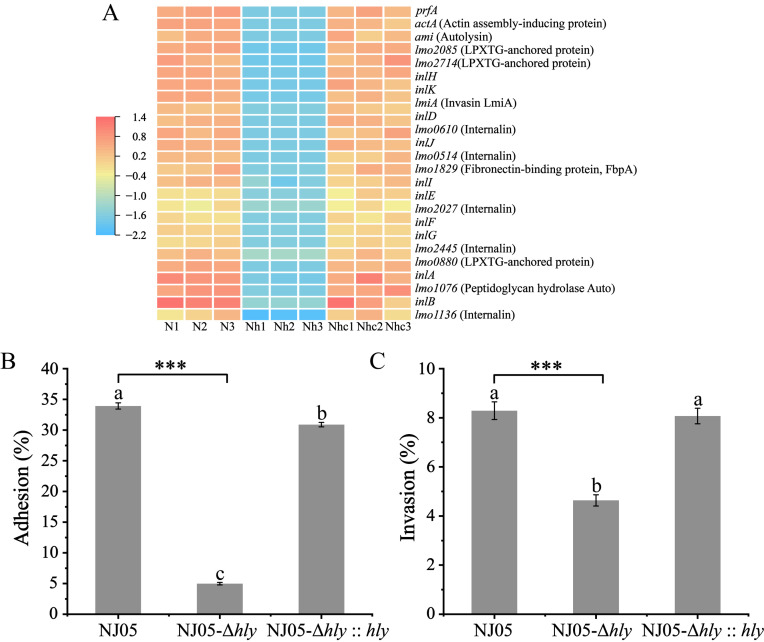
LLO-mediated adhesion and invasion of mutant NJ05-Δ*hly*. (**A**) Heatmap analysis of the expression of virulence-related genes. The legend for the diagram is shown in Figure 1. LLO-mediated adhesion (**B**) and invasion (**C**) of mutant NJ05-Δ*hly* to RAW264.7 cells following treatment with WT strain or mutant NJ05-Δ*hly*. Different letters in the figure indicate significant differences (*p* < 0.05, one-way ANOVA followed by Waller-Duncan). *** *p* < 0.001 when compared with the wild-type NJ05.

Following the deletion of *hly*, 18 genes encoding internalin were downregulated, along with those involved in bacterial autolysis (*auto* and *ami*) (Supplementary Figure S4 and Figure 4A). The results of experiments assessing the adhesion and invasion rates of macrophages confirmed these findings. The deletion of *hly* decreased the adhesion and invasion capabilities of *L. monocytogenes*. The adhesion and invasion rates of the wild-type strain NJ05 in RAW264.7 cells were 33.5% ± 0.52% and 8.25% ± 0.36%, respectively. In contrast, the strain NJ05-Δ*hly* exhibited markedly lower rates of 5.05% ± 0.21% and 4.63% ± 0.23%, respectively. When compared to the wild-type strain, the adhesion and invasion abilities of the mutant strain NJ05-Δ*hly* were decreased by 84.93% and 43.88%, respectively (*p* < 0.001) (Figure 4B,C).

### 3.6. Reduction in the EOP of L. monocytogenes After Infection with Mutant NJ05-Δhly

**Figure 5:**
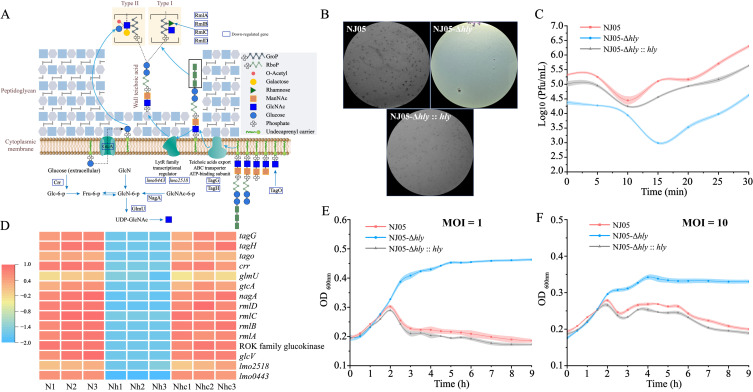
Susceptibility of phage vB-LmoM-NJ05 infected with mutants NJ05-Δ*hly*. (**A**) Biosynthetic pathways of WTA in *L. monocytogenes*. (**D**) Heatmap analysis of wall teichoic acid-related gene expression. The legend for the diagram is shown in Figure 1. (**B**) Plaques and (**C**) absorption effect of vB-LmoM-NJ05 phage on *L. monocytogenes* strains. (**E**,**F**) The lytic ability of vB-LmoM-NJ05 against mutants NJ05-Δ*hly* was reduced at MOI 1 and MOI 10. At an MOI of 10, the lytic ability was enhanced slightly, although the lysis effect was weaker than that of wild-type NJ05.

The major genes involved in the synthesis of wall teichoic acid (WTA) in *L. monocytogenes*, such as *tagG*, *tagH*, *tagO*, *crr*, *glmU*, *gtcA*, *glcV,* and *lmo0443*, were all downregulated. The absence of *hly* led to alterations in WTA, which may ultimately result in decreased susceptibility to phages (Figure 5A,D). Hence, relevant tests for phage susceptibility were conducted.

#### 3.6.1. Reduction of EOP in the Mutant NJ05-Δhly

EOP serves as an indicator of host susceptibility and the interaction between the host and phages. The phage susceptibility of mutant NJ05-Δhly to phage vB-LmoM-NJ05 was assessed using an EOP assay. The 76.92-fold reduction in EOP suggests the involvement of hly in certain aspects of the phage life cycle or its ability to penetrate bacterial cells. Thus, LLO may facilitate phage entry or replication (Table 2). In addition, phage plaques formed by the mutants NJ05-Δhly were smaller and less distinct than those produced by the parental strain (Figure 5B). This reduction in EOP shows that deletion of hly considerably affects the susceptibility of the mutants to the phage.

#### 3.6.2. Enhanced Phage Adsorption and Lysis Activity on the LLO Mutant

The observations demonstrated that the number of free phages against NJ05 decreased from 5.32 ± 0.04 to 4.38 ± 0.07 log (PFU/mL) after 10 min of adsorption. Strikingly, the adsorption of NJ05-Δ*hly* mutants was enhanced after infection with phage vB-LmoM-NJ05; free phages decreased from 4.38 ± 0.08 to 2.97 ± 0.04 after 15 min of incubation (Figure 5C). The in vitro lytic activity of the phage against *L. monocytogenes* cultures was evaluated via challenge assays using varying phage concentrations (MOIs) to monitor bacterial elimination over time. Phage concentrations at MOIs of 1 and 10 effectively inhibited the growth of both wild-type and defective mutants within 9 h (Figure 5E,F). The lytic trends of NJ05 and NJ05-Δ*hly*::*hly* were similar. It exhibited slight growth within the first two hours, followed by gradual suppression by the phage. However, the defective strain NJ05-Δ*hly* gradually increased during the first 4 h and subsequently maintained a certain concentration. The data suggest that while phages effectively suppressed the growth of wild-type NJ05 and the complemented strain NJ05-Δ*hly*::*hly*, their inhibitory effect on NJ05-Δ*hly* was significantly reduced (Figure 5E,F). In summary, the absence of *hly* reduced the lytic activity of this phage.

### 3.7. Reduction in Phage-Mediated Biofilm Removal of the LLO Mutant

**Figure 6:**
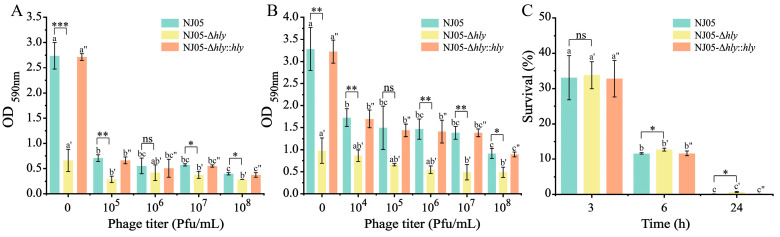
Effect of phages on biofilm formation and removal. (**A**) Biofilm inhibition and (**B**) removal activities of vB-LmoM-NJ05 phages for NJ05, NJ05-Δ*hly*, and NJ05-Δ*hly*. (**C**) Survival of *L. monocytogenes* strains in biofilms after vB-LmoM-NJ05 phage treatment. Different letters in the figure indicate significant differences and the letters with primes indicate different groups (*p* < 0.05, one-way ANOVA followed by Waller-Duncan). * *p* < 0.05; ** *p* < 0.01; *** *p* < 0.001 when compared with the wild-type NJ05; ns, no significance.

The biofilm-forming ability of strain NJ05-Δ*hly* declined following deletion of *hly*. Phages were added to the culture of the NJ05-Δ*hly* strain after 24 h of incubation. At a concentration of 10^5^ PFU/mL, the phage effectively inhibited biofilm formation, demonstrating a strong inhibitory effect on NJ05-Δ*hly* (Figure 6A). Furthermore, an increase in phage concentration (10^6^–10^8^ PFU/mL) positively correlated with enhanced inhibition of biofilm formation. Phages at a concentration of 10^8^ PFU/mL almost completely removed the biofilms formed by NJ05-Δ*hly*, in contrast to the results observed for the wild-type strain NJ05 (Figure 6B). These observations establish that the absence of *hly* debilitates biofilm-forming capability, thereby augmenting the efficacy of phage-mediated biofilm removal.

Furthermore, the survival rates of the strains in the biofilms were assessed after adding phage vB-LmoM-NJ05. After 3 h of treatment, the survival rates of the mice treated with NJ05, NJ05-Δ*hly,* and NJ05-Δ*hly*::*hly* were 33.33% ± 6.26%, 34.12% ± 3.83%, and 32.80% ± 5.22%, respectively. After 24 h of exposure, these rates decreased significantly to 0.10% ± 0.04%, 0.68% ± 0.16%, and 0.10% ± 0.02%, respectively (Figure 6C). These findings confirm that phage vB-LmoM-NJ05 substantially inhibits the growth of *L. monocytogenes* in biofilms, with a linear relationship observed between treatment duration and survival rate reduction.

### 3.8. Mitigation of L. Monocytogenes-Induced Host Cell Damage by Phages

**Figure 7:**
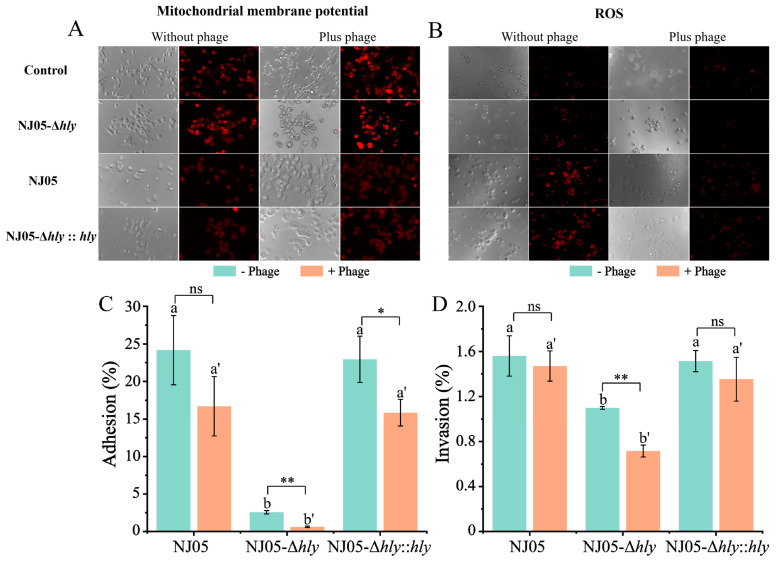
Phage vB-LmoM-NJ05 alleviates the cell damage caused by *L. monocytogenes* strains. RAW264.7 cells were incubated with 10^8^ PFU/mL phages vB-LmoM-NJ05 in advance for prevention for 24 h. The cells were then washed thrice with PBS and infected with *L. monocytogenes* strains at an MOI of 50 for approximately 1.5 h. (**A**) Damage to the cell mitochondria was reduced due to pre-treatment with the phage. (**B**) Accumulation of ROS in the cell mitochondria decreased after treatment with the phage in advance. Cell damage and ROS superoxide levels were analyzed using fluorescence microscopy. Phage vB-LmoM-NJ05 could restrain the adhesion (**C**) and invasion (**D**) of *L. monocytogenes* strain. RAW264.7 cells were incubated with 0.1 mL of phage vB-LmoM-NJ05 (10^8^ PFU/mL) for 24 h. The cells were then washed and infected with Lm at an MOI of 100 for approximately 1.5 h. Viable bacteria were enumerated and used to evaluate adhesion and invasion. Different letters in the figure indicate significant differences and the letters with primes indicate different groups (*p* < 0.05, one-way ANOVA followed by Waller-Duncan). * *p* < 0.05; ** *p* < 0.01 when compared with the wild-type NJ05; ns, no significance.

Compared to the wild-type strain NJ05, the defective strain NJ05-Δhly caused significantly lower mitochondrial damage in RAW264.7 cells after 1.5 h of infection. Therefore, the mitochondrial membrane potential of cells infected with the NJ05-Δhly strain did not differ from that of the control cells. Similarly, the accumulation of ROS in RAW264.7 cells infected with the NJ05-Δhly strain was correspondingly reduced, indicating a considerable decrease in virulence. Furthermore, pre-treatment with vB-LmoM-NJ05 offered marked protection against mitochondrial damage inflicted by wild-type NJ05, as inferred from the reduction in membrane potential (Figure 7A). Concurrently, the accumulation of ROS in the mitochondria decreased (Figure 7B). These findings imply that phage vB-LmoM-NJ05 effectively protects mitochondrial integrity from the detrimental effects of *L. monocytogenes*.

### 3.9. Mitigation of Adhesion and Invasion Abilities of NJ05-Δhly to Cells by Phages

Figure 7C,D illustrate that the deletion of *hly* significantly impaired the adhesion and invasion capabilities of NJ05-Δ*hly* in RAW264.7 macrophages. When exposed to the phage vB-LmoM-NJ05 at a concentration of 10^8^ PFU/mL, the adhesion rate of wild-type NJ05 decreased from 24.38% ± 4.61% to 16.88% ± 3.96%, resulting in an inhibition rate of 30.76%. In comparison, the adhesion rate of the NJ05-Δ*hly* strain reduced from 2.66% ± 0.22% to 0.75% ± 0.10%, indicating a higher inhibition rate of 71.80% (*p* < 0.01). The invasion rate of the wild-type strain declined slightly from 1.58% ± 0.18% to 1.48% ± 0.13%, reflecting a 6.33% decrease. Conversely, the invasion rate for the NJ05-Δ*hly* strain diminished from 1.11% ±0.01% to 0.73% ± 0.05%, resulting in an inhibition rate of 34.23% (*p* < 0.01). These observations imply that vB-LmoM-NJ05 considerably reduces the adhesion and invasion capabilities of *L. monocytogenes* in RAW264.7 macrophages, with the effect being particularly pronounced in the defective strain NJ05-Δ*hly* (Figure 7D).

## 4. Discussion

*L. monocytogenes* is a facultative intracellular pathogen that can actively invade mammalian cells and replicate within them [34]. The pathogen avoids residing within the internalization vacuole by secreting the pore-forming toxin LLO and two phospholipases (PlcA and PlcB), which disrupt the vacuolar membrane, enabling its translocation to the host cell cytoplasm [35]. In this study, the susceptibility of *L. monocytogenes* to phages markedly decreased in the absence of LLO. However, in contrast to previous reports, LLO did not affect phage adsorption efficiency. Furthermore, the lack of LLO impacted biofilm formation by *L. monocytogenes*. The virulence properties of adhesion and invasion of these mutants were significantly reduced in the defective strain NJ05-Δ*hly*, decreasing by approximately 85% (*p* < 0.001).

Numerous pathogens, including viruses and bacteria, target mitochondria to disrupt the apoptotic machinery of host cells [36,37]. During infection, *L. monocytogenes* can profoundly alter mitochondrial dynamics and host cell mitochondrial morphology, inducing rapid and drastic fragmentation of the mitochondrial network [38]. The secreted pore-forming toxin LLO is the primary bacterial factor that disrupts the mitochondrial network and impairs mitochondrial function. Specifically, LLO dissipates the mitochondrial membrane potential, accompanied by a reduction in respiratory activity and cellular ATP levels. Conversely, the defective strain NJ05-Δ*hly* inflicted reduced mitochondrial damage in RAW264.7 macrophages, which is consistent with previous studies [35]. Furthermore, mitochondrial ROS are key components of the innate immune response against intracellular bacteria and contribute to the bactericidal activity of macrophages [39]. In this study, NJ05-Δ*hly* significantly alleviated the accumulation of mitochondrial ROS. The phage vB-LmoM-NJ05 protects mitochondria from damage caused by *L. monocytogenes*, although the mechanisms linking phage activity to mitochondrial ROS generation remain to be elucidated.

*L. monocytogenes* can persist in the food industry by forming biofilms [40]. The investigation of the role of *hly* in *L. monocytogenes* biofilm formation demonstrated the complex interplay between genetic factors and environmental conditions influencing the pathogenicity of the bacterium. The findings of Pang underscore the remarkable adaptability of *L. monocytogenes* to refrigerated environments, where it thrives and forms biofilms that confer resistance to disinfectants [41]. This adaptability likely aids its survival in food-associated environments and its transmission to humans [6]. Virulent phages of *Listeria* are effective against a broad range of species and may help reduce the occurrence of *Listeria*-associated food recalls by preventing its contamination [13]. The present study found that the biofilm-forming ability of NJ05-Δ*hly* was significantly lower than that of parental NJ05. Furthermore, biofilm formation by NJ05-Δ*hly* was not directly related to temperature, BHI, or NaCl concentration. Interestingly, increasing the glucose concentration to 8% substantially augmented biofilm formation (*p* < 0.01), suggesting that glucose is a necessary nutrient or energy source [42].

The use of *Listeria* phages is a potential antimicrobial strategy in the food industry because of their ability to eliminate pathogenic bacteria [13,43,44]. The biology of phage infection has been extensively investigated, beginning with phage attachment to the host surface via binding to suitable receptors. The location and type of bacterial cell surface receptors can vary immensely, from cell wall teichoic acids and lipopolysaccharides to flagellar proteins [45,46,47]. Host surface recognition of *Listeria* involves carbohydrates, such as teichoic acids, covalently linked to peptidoglycan cell walls [48]. Receptor binding is vital for initiating infection. A recent study on *L. monocytogenes* reported that specific glycosyl modifications on wall-associated glycopolymers (WTA) are involved in phage adsorption and the retention of the major virulence factor InlB [49]. In the present investigation, phage susceptibility was considerably altered in the LLO-defective strain NJ05-Δ*hly*, with an over 70-fold reduction in EOP. Therefore, LLO may be a contributing factor in influencing susceptibility to phages. Phage adsorption is the initial step in infection, and the decreased susceptibility of the defective strain is likely linked to this process. Notably, the adsorption capacity of the NJ05-Δ*hly* strain increased slightly, although the overall trend was similar to that of the wild-type strain. However, the lytic activity was substantially reduced compared to that of the parental strains. Similarly, the reduction in antibacterial efficacy affected the biofilm removal capabilities. Furthermore, vB-LmoM-NJ05 effectively attenuated the adhesion and invasion capabilities of *L. monocytogenes* in RAW264.7 cells and exhibited more pronounced activity against the defective strain NJ05-Δ*hly*.

The interaction mechanisms between LLO and phages were further examined using transcriptomic analysis of the defective strain NJ05-Δ*hly*. In comparative transcriptome profiling, 322 genes were significantly upregulated and 1896 were significantly downregulated. Genes associated with N-acetylglucosamine synthesis, such as *glmU*, *glcV*, *galE*, *cps4I*, *nagA*, *pdgA*, and *murA*, were significantly downregulated. This finding suggests that the absence of *hly* alters the WTA composition of *L. monocytogenes* [50]. The considerable decrease in phage infectivity could be attributed to the expression of surface receptors, which may also influence phage interactions [16,51]. LLO may aid InlA and InlB in the adhesion and invasion processes of *L. monocytogenes* in host cells. As a result, virulence-related genes, such as *inlA*, *inlB*, *inlF*, and *inlJ*, were significantly downregulated, leading to altered invasion and biofilm formation capabilities [52,53]. In addition, the expression of genes associated with biofilm formation was considerably decreased in the absence of LLO. Interestingly, motility was considerably enhanced, potentially achieved via the upregulation of flagellar motility-associated genes. The interaction among the complex proteins (*fliG*, *fliM*, *fliY*, and *fliN*), which constitute the flagellar motor switch, and the *cheY* gene induces an alteration in flagellar rotation from clockwise to counter-clockwise [31,32], facilitating a shift from tumbling to smooth-swimming behavior [33]. The expression of 15 of the 26 genes involved in flagellar assembly was upregulated in the mutant NJ05-Δ*hly*. These genes include flagellar motor switch regulatory genes (*fliM*, *fliY*, and *fliN*) and motility genes (*motA*). This upregulation could be ascribed to a decrease in c-di-GMP levels owing to the absence of *hly* [54]. The lack of this gene inhibits the synthesis of polysaccharide matrices and adhesins, reducing biofilm adhesion rates while enhancing strain motility.

In osmotically stabilized environments, *L. monocytogenes* can evade phage predation by transiently converting to the L-form, a cell wall-deficient state [55]. This escape is triggered when endolysins disintegrate the cell wall externally, resulting in the turgor-driven extrusion of viable L-form cells that lack cell walls. This study identified that deletion of *hly* promoted this transient conversion. These findings suggest that such a conversion may help evade complete eradication by phage attacks.

## 5. Conclusions

Food recalls owing to *L. monocytogenes* are common and associated with substantial economic costs. In food-processing environments, *Listeria* species inhabit biofilms, bacterial structures that shield them from environmental stressors, and are often adhered to surfaces. The significance of our work lies in the novel observation that deletion of *hly* alters phage susceptibility. Although LLO does not directly interact with phages as a receptor, it indirectly regulates the expression of related receptors, serving as an indirect receptor for phages. These findings emphasize the vital role of virulence factors in phage applications and open novel avenues for the potential use of phages in the development of biosanitizers.

## Figures and Tables

**Table 1:** PCR primers used in this study.

Gene Name	Primer Sequence (5′-3′)	Product Length
*hly*-F1	AAGTCCTAAGACGCCAATC	1345
*hly*-R1	TTACCGTTCTCCACCATTC	
*hly*-F2	GTGGAGGCATTAACATTTGT	400
*hly*-R2	CTATAGGTGGCTTAAACTTTGG	
*hly*-F3	TAACGACGATAAAGGGACAG	1961/372
*hly*-R3	GGCTTAAACTTTGGGATATGC	

**Table 2:** EOP of mutants NJ05-Δ*hly* infected by phage vB-LmoM-NJ05.

Strain	Titer (PFU/mL)	EOP
NJ05	(4.2 ± 0.02) × 10^8^	1.0
NJ05-Δ*hly*	(5.46 ± 0.01) × 10^6^	0.013
NJ05-Δ*hly*::*hly*	(3.83 ± 0.04) × 10^8^	0.913

## Data Availability

The original contributions presented in the study are included in the article/Supplementary Material, further inquiries can be directed to the corresponding authors.

## Supplementary Materials

The following supporting information can be downloaded at: https://www.mdpi.com/article/10.3390/foods14152554/s1, Figure S1. Hemolytic activities of *L. monocytogenes* NJ05, NJ05-Δ*hly* and NJ05-Δ*hly*::*hly* on sheep blood agar plate; Figure S2. Volcanic map of differentially expressed genes; Figure S3. The lifecycle of biofilm formation; Figure S4. The downregulated genes primarily pertain to the zipper mechanism (*inlA*, *inlB*) and autolysins (*auto*, *ami*) modulate bacterial invasion.
