# Supplementary material for: The Virulence Factor LLO of Listeria monocytogenes Can Hamper Biofilm Formation and Indirectly Suppress Phage-Lytic Effect
Source: Foods. 2025 Jul 22;14(15):2554. doi: 10.3390/foods14152554 (PMC12346585; doi:10.3390/foods14152554)
Supplement: Supplementary file 1 [file foods-14-02554-s001.zip › foods-3740151-supplementary.pdf]

Supplementary material

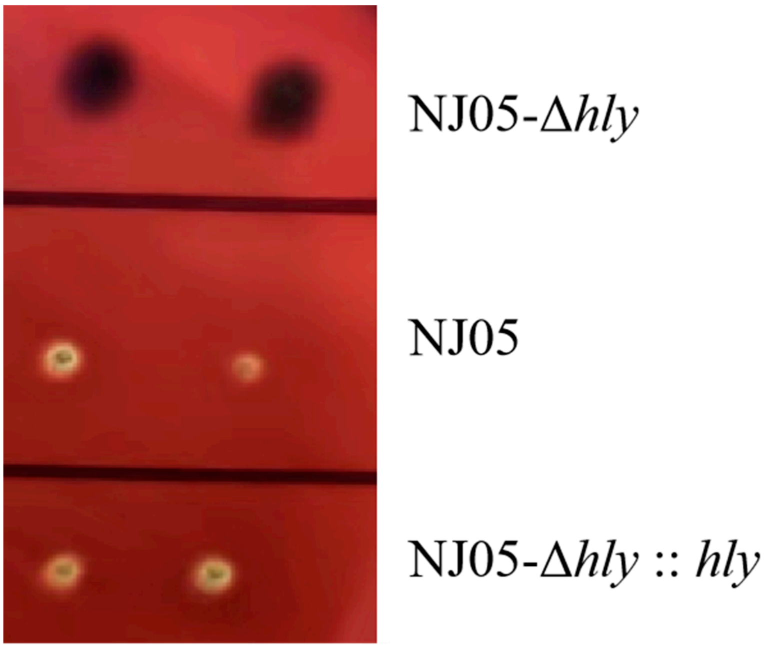

Figure S1. Hemolytic activities of L. monocytogenes NJ05, NJ05- △ hly and NJ05- △ hly::hly on sheep blood agar plate

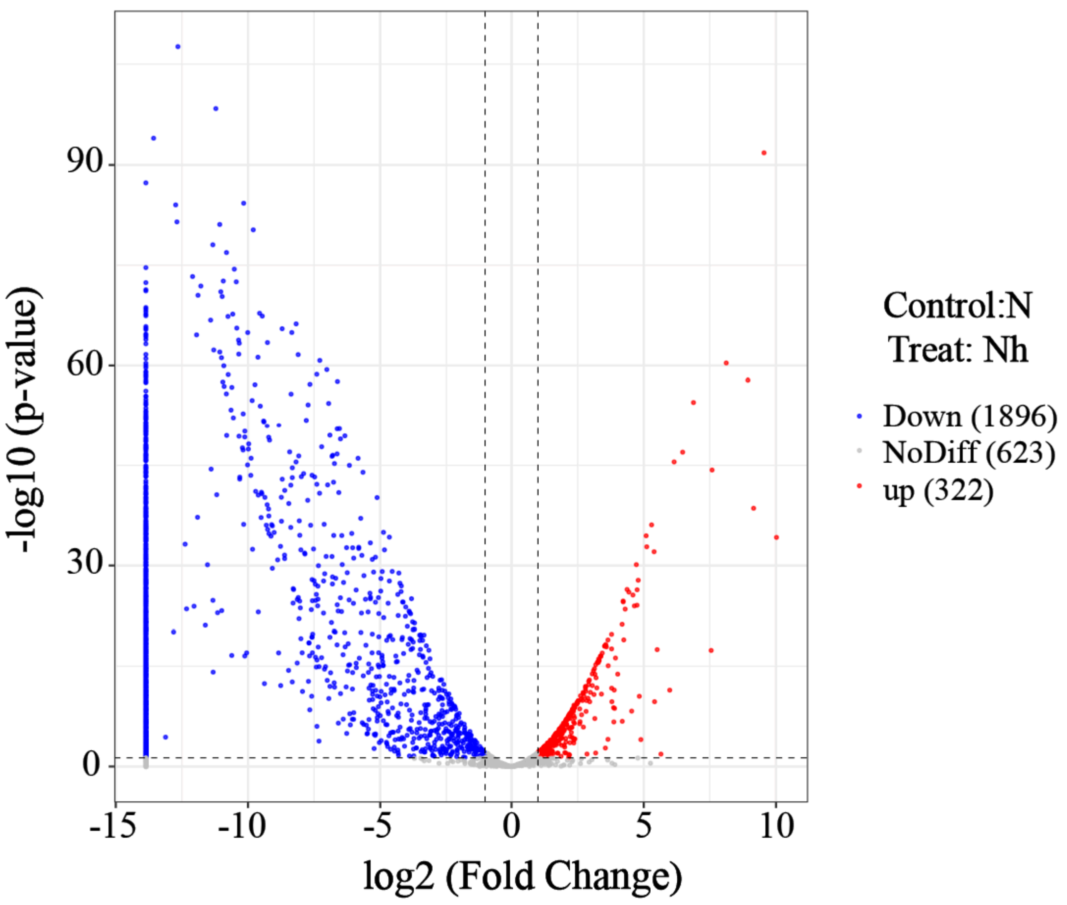

Figure S2. Volcanic map of differentially expressed genes

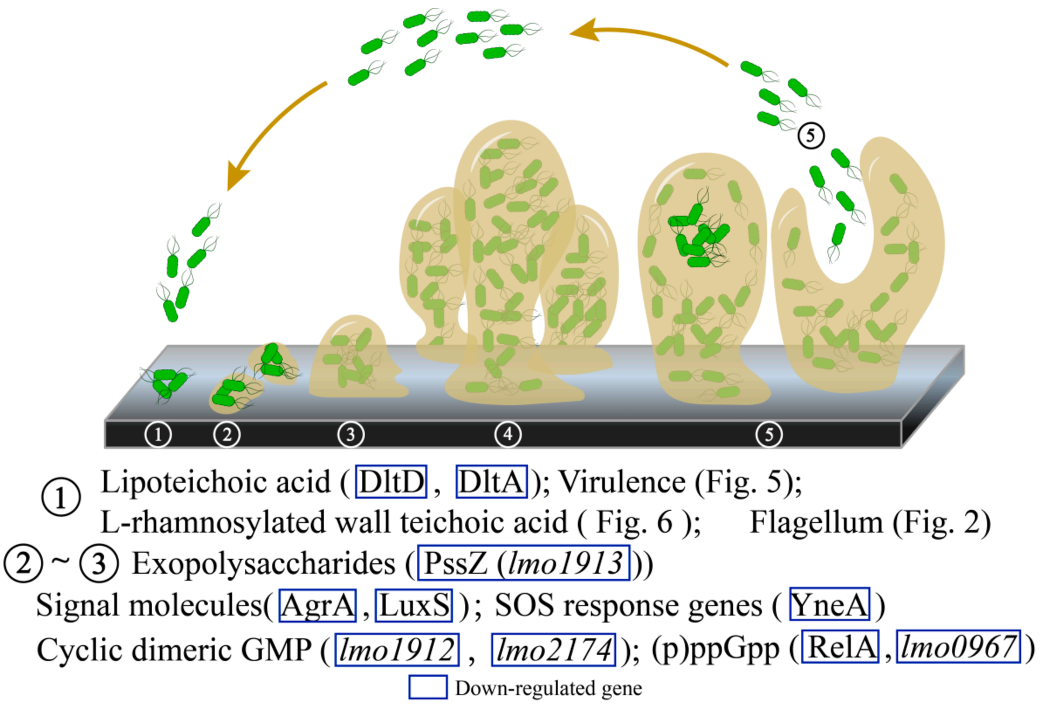

Figure S3. The lifecycle of biofilm formation: The downregulated genes primarily pertain to the attachment stage, encompassing genes responsible for lipoteichoic acid synthesis (*dltD*, *dltA*), flagellar genes (Fig. 2B), virulence genes (Fig. 3B), and L-rhamnosylated wall teichoic acid (Fig. 6B). During the maturation stage, the downregulated genes were concerned with regulatory genes for exopolysaccharides and ppGpp (*pssZ*, *relA*). In addition, regulatory genes for some signal molecules (*agrA*, *luxS*, and *cGMP*) were also downregulated.

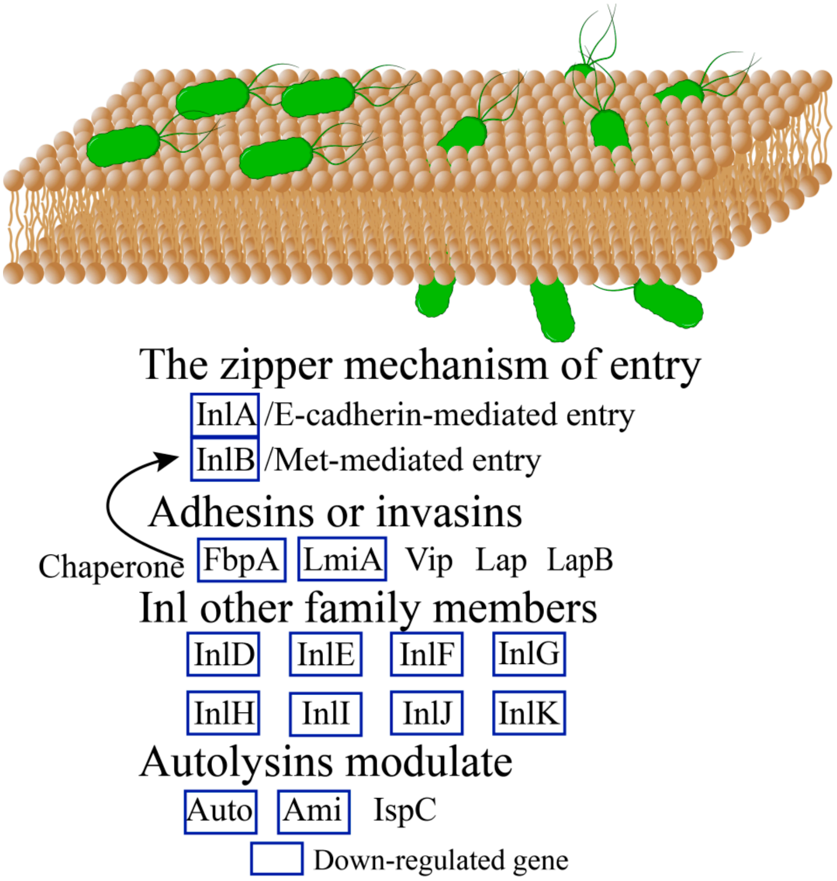

Figure S4. The downregulated genes primarily pertain to the zipper mechanism (*inlA*, *inlB*) and autolysins (*auto*, *ami*) modulate bacterial invasion. In addition, regulatory genes associated with other members of the internalin family were found to be downregulated.
